# Giant aneurysm of the left main coronary artery with fistulous communication to the right atrium

**DOI:** 10.1186/s13019-015-0324-8

**Published:** 2015-09-11

**Authors:** Zhicheng Zhu, Yong Wang, Rihao Xu, Dan Li, Tiance Wang, Bo Li, Shudong Zhang, Kexiang Liu

**Affiliations:** Department of Cardiovascular Surgery, The Second Hospital of Jilin University, Changchun, 130041 P.R. China

**Keywords:** Coronary artery aneurysm, Coronary artery fistula, Surgical treatment

## Abstract

The giant coronary artery aneurysm combined with coronary artery fistula is extremely uncommon. In our case, there was a giant aneurysm of the left main coronary artery with fistulous communication to the right atrium, combined with moderate aortic valve regurgitation, which was initially found by transthoracic echocardiogram and subsequently confirmed by the 256-slice multidetector computer tomography (MDCT) coronary angiography. After consultation, the patient received surgical treatment, including the closure of the drainage and origin sites of the aneurysm and the aortic valve replacement. The patient recovered uneventfully.

## Background

Coronary artery aneurysm (CAA) is defined as dilation of a coronary artery 1.5 times the adjacent normal segment. Only more than 4 cm in diameter of coronary artery aneurysms, can be called giant coronary artery aneurysms [1]. CAA represent anomalies identified in 0.15–4.9 % of patients undergoing coronary angiography, which combined with coronary artery fistula is extremely uncommon. The etiology of CAA involves congenital malformation, chest trauma, atherosclerosis, systemic lupus erythematosus, Behcet’s disease, Kawasaki disease, sarcoidosis, directional coronary atherectomy and postimplantation of a drug-eluting stent. A giant coronary artery aneurysm has poor prognosis, which may cause thromboembolism, aneurysm rupture or hemodynamic abnormalities due to compression [2]. Therefore, making earlier diagnosis and treatment are very important. Owing to the rarity of giant CAAs, no standard treatment protocol has been established. Here we present the patient with a giant aneurysm of the left main coronary artery with fistulous communication to the right atrium, combined with moderate aortic valve regurgitation. After surgery teartment, the patient recovered uneventfully.

## Case presentation

**Figure 1:**
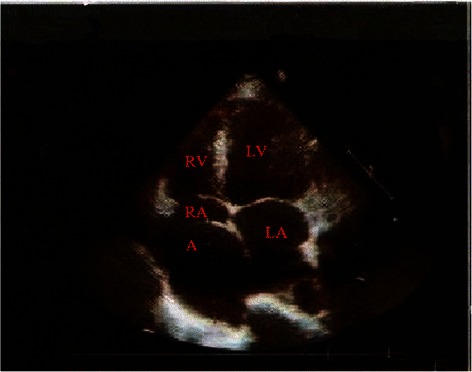
Preoperative UCG shows the aneurysm between the left atrium and right atrium. Abbreviations LV: Left ventricle; LA: Left Atrium; RV: Right ventricle; RA: Right Atrium; A: Aneurysm

**Figure 2:**
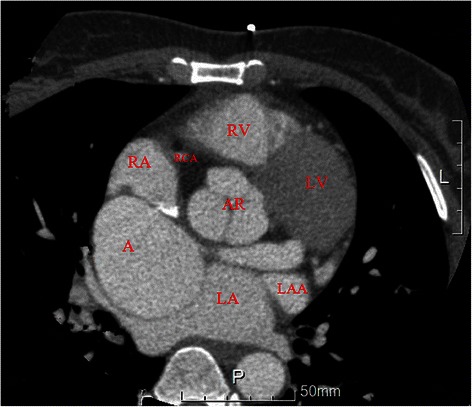
CTA coronary angiography showed the relation between aneurysm and left main trunk. Abbreviations LV: Left ventricle; LA: Left Atrium; RV: Right ventricle; RA: Right Atrium; A: Aneurysm; RCA: Right Coronary artery; LAA: Left Atrium Appendix; AR: Aortic Root

**Figure 3:**
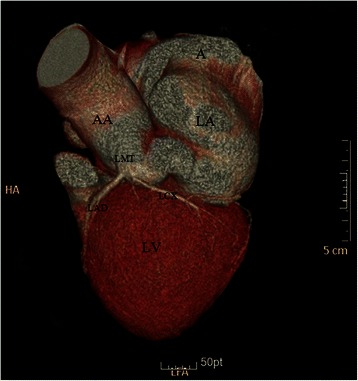
3D-reconstruction of coronary CT demonstrated the positional relation of the coronary aneurysm and surrounding structures. Abbreviations LV: Left ventricle; LA: Left Atrium; A: Aneurysm; AA: Ascending Aorta; LMT: Left Main Trunk; LAD: Left Anterior Descending; LCX: left circumflex coronary artery

A 48-year-old woman was admitted with complaints of chest discomfort and shortness of breath for 10 years, paroxysmal nocturnal dyspnea in last 10 days. On physical examination, her blood pressure was 128/90 mmHg. Auscultation reveals a continuous murmur over her left upper sternum border (Levine 3/6), and an early diastolic murmur that radiates toward the apex of the heart. The chest X-ray revealed cardiomegaly with cardiothoracic ratio 61.0 %. The electrocardiogram (ECG) showed a sinus rhythm with no significant ST-T changes. The transthoracic echocardiogram demonstrated the dilation of the left ventricle, a giant saccular aneurysm arose from a dilated left main trunk, which oppressing the right atrium and superior vena cava, color Doppler examination visualized a possible communication with the right atrium and moderate aortic valve regurgitation (Jet width 54 % of LVOT, AR area 7.2 cm^2^) (Figure 1). To further evaluation, The multidetector computer tomography (MDCT) coronary angiography was performed, the reconstructions of 3D volume-rendered (VR) images clearly showed a dilated tortuous left main trunk (LMT) (20 mm in diameter) arose from the dilated left coronary sinus. The LMT coursed behind the aortic root with tortuosity, and it was transformed into a giant thin-walled saccular aneurysm (60 mm*58 mm) between the left and right atrium, and a fistula to the right atrium near the superior vena cava-right atrium junction (Figure 2, 3). There is no significant lesions in all coronary arteries.

**Figure 4:**
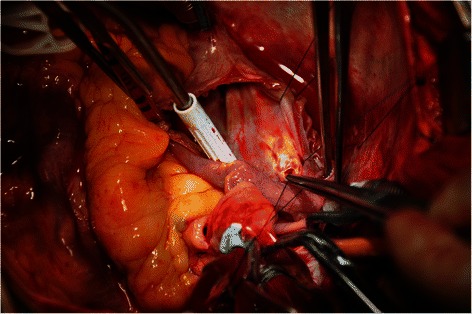
Intraoperative image shows that the fistula to the right atrium was found

Due to its risk of rupture and compression of the heart, surgical repair of the aneurysm was recommended. After consultation, the patient underwent elective open-heart surgery through median sternotomy and extracorporeal circulation. The aorta was cross-clamped, and heart was arrested with antegrade and retrograde cardioplegia. The aneurysm between left and right atrium was clearly identified. Once the right atrium opened, a fistulous path to the right atrium of 0.5 mm in diameter was found at the top of atrial septum (Figure 4). The aneurysm is opened vertically from the interatrial groove, the connection with the left main trunk was a narrow path of 0.5 mm in diameter. The two fistulas of the aneurysm on the atrial septum and LMT were closed by 5–0 polypropylene with pledgets, and the incision of aneurysm was closed with 4–0 polypropylene suture. The aortic valve was replaced with a 21 mm St. Jude mechanical regent valve prosthesis.

**Figure 5:**
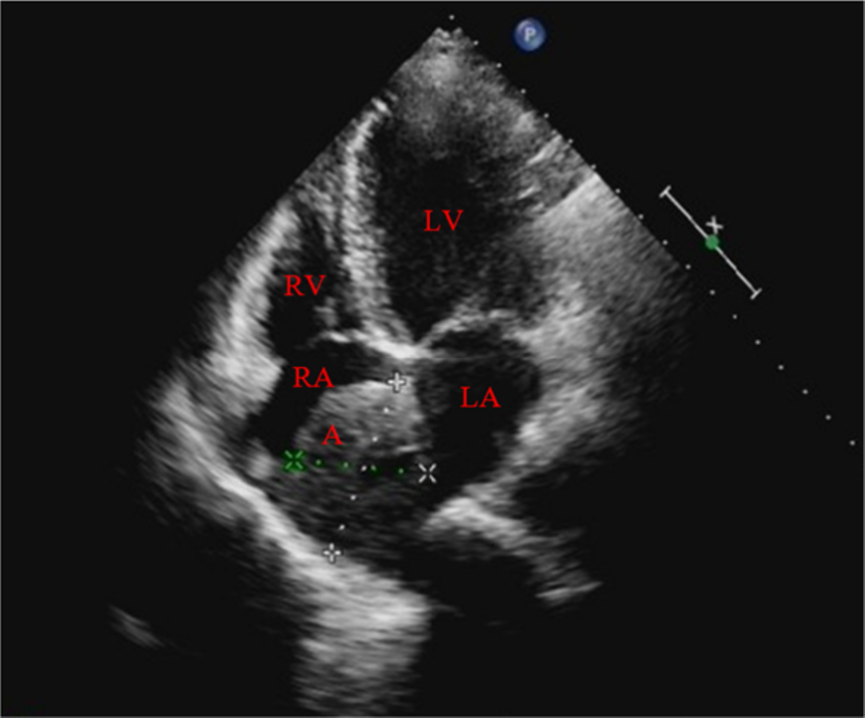
Postoperative UCG shows residual tumor, the proportion of LA and RA improved. Abbreviations LV: Left ventricle; LA: Left Atrium; RV: Right ventricle; RA: Right Atrium; A: Aneurysm

**Figure 6:**
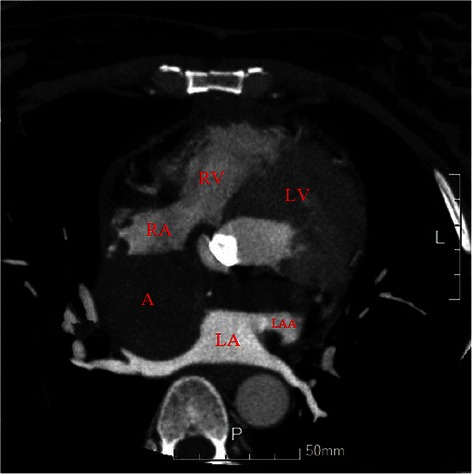
Postoperative CTA coronary angiography showed the thrombosis of aneurysm and fistulous connection. Abbreviations LV: Left ventricle; LA: Left Atrium; RV: Right ventricle; RA: Right Atrium; A: Aneurysm; LAA: Left Atrium Appendix

In this case, it is not possible to determine whether the aneurysm is secondary to fistula since progressive history was not followed up. However, it is reasonable to speculate that the aneurysm was secondary to fistula. Analysis of the imaging of the CTA, the progressive AR could have been induced by aortic valve degeneration which may not be associated with the fistula. The function of left main stem aneurysm has not been affected by this aneurysm, and the dilated LV was induced by progressive AR which was in turn caused by the dilated left coronary sinus. The postoperative CVP was 6 cm H2O which did change significantly compared with that preoperatively. This finding supports the above notion of the disease process in our patient. Postoperative transthoracic echocardiogram and MDCT of coronary artery demonstrated the thrombosis of aneurysm and the absence of fistulous connection (Figure 5, 6). The patient recovered uneventfully, and her subjective symptom disappear entirely.

## Conclusions

Coronary artery aneurysm (CAA) is defined as dilation of a coronary artery 1.5 times the adjacent normal segment. Only more than 4 cm in diameter of coronary artery aneurysms, can be called giant coronary artery aneurysms [1]. Coronary artery aneurysm localized in the right coronary artery (RCA) most frequently, secondly localized in left anterior descending (LAD) and left circumflex (LCX), and only exceptionally in the left main trunk. The aneurysmal sac may be fusiform or saccular, the former is the most common type and mostly related with atherosclerosis, the latter is more easily to rupture, thrombosis or fistula formation [3]. CAA is not common, which is present in 0.3–4.9 % of the general population [3]. In recent years, the incidence of CAA has increased, probably due to the use of coronary angiography and multi-detector CT angiography. It is a rare entity that the giant CAA combined with coronary artery fistula. Yu et al. [4] reported that CAA was found in 5.9 % of patients with congenital coronary artery fistula, which was found in only 0.2 % of patients who had undergone open heart surgery. To the giant aneurysm, which arose form the left main trunk and located in the atrial septum, there has never been reported in the literature.

Owing to the rarity of giant CAAs, there is no standard treatment protocol that has been established. Without doubt, large, saccular, rapidly growing or symptom-causing aneurysms are indicators for open-heart surgery. The coronary aneurysm impacts the hemodynamics and reduces the distal perfusion of coronary artery which results in decreased coronary flow and leads to progressive dilation of the left main trunk. In this case, the dilated left sinus increased the risk of aortic root rupture, so the surgery was necessary. The alteration of hemodynamics of this disease is left-to-right effect which could affect the function of right heart. For a giant coronary artery aneurysm with a clear oppression of the heart, resection of the coronary artery aneurysm and coronary artery bypass grafting are recommended. However, recent reports have demonstrated the advantage of trans-catheter closure of coronary artery fistula (CAF) by using occlusion devices with challenging results as alternative option to surgery [5]. In our case, the open-heart surgery was necessary because the giant aneurysm and the aortic valve regurgitation coexisted. To ensure myocardial protection, we incised right atrium to block the fistula, and cardioplegia perfused combined antegrade with retrograde cardioplegia perfusion. Coronary artery bypass grafting was not necessary since the left main stem integrity was intact. The aneurysm was excised by the neck from the left main stem. Here we took a different surgical approach. The proximal and distal fistula was closed by running suture which did not affect the coronary flow and avoided CABG surgery compared with the approach reported by Margux and colleagues [6]. Trans-catheter occlusion with embolization reported by Wang et al. [7] is another therapeutic choice for this disease, but the AR in our patient made the trans-catheter occlusion procedure unsuitable.

The patient recovered uneventfully. Postoperative MDCT of coronary artery showed the thrombosis of aneurysm and the absence of fistulous connection. Finally, we recommend our surgical intervention as a effective treatment for giant CAA with coronary artery fistula.

## Consent

Written informed consent was obtained from the patients for publication of this case report and all accompanying images.

## Abbreviations

CAA: Coronary artery aneurysm
MDCT: Multidetector computer tomography
LMT: Left main trunk
LVOT: Left ventricular outflow tract

## References

[1] Nichols L, Lagana S, Parwani A (2008). Coronary artery aneurysm: a review and hypothesis regarding etiology. Arch Pathol Lab Med.

[2] Panzer J, De Jaeger A, Suys B (2008). Rupture of giant coronary arterial aneurysm without progressive dilation. Cardiol Young.

[3] Dodge-Khatami A, Mavroudis C, Backer CL (2000). Congenital heart surgery nomenclature and database project: anomalies of the coronary arteries. Ann Thorac Surg.

[4] Yu W, Yusa L, Shou H, Wei P, Tao Q (2001). Surgical treatment of giant coronary artery aneurysm. Asian Cardiovasc Thorac Ann.

[5] Kamiya H, Yasuda T, Nagamine H, Sakakibara N, Nishida S, Kawasuji M (2002). Surgical treatment of congenital coronary artery fistulas: 27 years’ experience and a review of the literature. J Card Surg.

[6] Margaux Pontailler MD, Didier Vilarem MD, Jean-Francois Paul MD, Philippe H, Deleuze MD (2015). Isolated huge aneurysm of the left main coronary artery in a 22-year-old patient with Type 1 neurofibromatosis. Ann Thorac Surg.

[7] Wang H, Luo X, Wang W, Wang X, Yang C, Zeng C (2012). Successful transcatheter patent ductus arteriosus occluder embolization of a congenital left coronay artery aneurysm and fistulas draining into the right atrium. Ann Thorac Cardiovasc Surg.

